# Reporting of Physicians’ or Investigators’ Choice of Treatment in Oncology Randomized Clinical Trials

**DOI:** 10.1001/jamanetworkopen.2021.44770

**Published:** 2022-01-21

**Authors:** Timothée Olivier, Alyson Haslam, Vinay Prasad

**Affiliations:** 1 Department of Oncology, Geneva University Hospital, Geneva, Switzerland; 2 Department of Epidemiology and Biostatistics, University of California, San Francisco

## Abstract

This cross-sectional study examines the prevalence of restricted choice of treatment in control groups among physicians and investigators conducting oncology randomized clinical trials (RCTs).

## Introduction

Randomized clinical trials (RCTs) aim to rigorously evaluate the benefits and risks of any intervention. However, RCTs may be limited if the control group does not reflect the ongoing standard of care, and especially if the control group is inferior to standard care. A cross-sectional analysis of 95 consecutive Food and Drug Administration approvals of anticancer agents between 2013 and 2018 showed that 16 (17%) were based on RCTs with suboptimal control groups.^1^

Theoretically, this limitation can be overcome if the physician is allowed to choose the control-group treatment with access and freedom. Increasingly, trials refer to a control group of physician’s choice or investigator’s choice, which may be perceived as a real (unrestricted) choice among all treatment options. However, this choice can be restricted and ambiguously reported with 2 consequences. First, when the choice is restricted to a limited number of options, the wording is misleading for physicians considering it was unfettered and unlimited. Second, the restriction may penalize the control group, making it substandard by not allowing all treatment options in a specific setting.

## Methods

This cross-sectional study adhered to Strengthening the Strengthening the Reporting of Observational Studies in Epidemiology (STROBE) reporting guideline. Because we used publicly available data, and this was not human subjects research, in accordance with 45 CFR §46.102(f), we did not submit this study to an institutional review board or require informed consent procedures.

We sought to determine the total number of published oncology RCTs reporting a physician’s (or investigator’s) choice, their evolution over time, whether the choice was real (unrestricted) or restricted. We systematically reviewed all reports on RCTs mentioning physician’s or investigator’s choice (eMethods and eFigure in the Supplement). To be included, articles had to report on RCTs studying anticancer intervention, mentioning “physician’s choice” or “investigator’s choice” within the title or the abstract. We excluded nononcology reports, phase 1 studies, commentaries, perspectives, and subsequent publication of the same trial. We did not restrict the period of inclusion. The analysis was descriptive, and frequencies were calculated for categorical variables throughout. Statistical analysis was performed using R software version 4.0.4 (R Project for Statistical Computing) on November 1, 2021.

## Results

Out of the 284 initially identified studies, 92 studies met our inclusion criteria, published between 2007 and 2021 (Table). Of these 92 oncology RCTs, the most common tumor type studied was breast cancer (22 of 92 RCTs; 23.9%). Over time, there was an increase in the number of published studies mentioning a physician’s or investigator’s choice in the control group of oncology RCTs (eg, 2 studies in 2007, 3 studies in 2014, and 12 studies in 2021). There were 82 industry-sponsored trials (89.1%) and 10 nonprofit sponsored trials (10.9%). Among the 82 industry-sponsored trials, there were 71 RCTs (77.2%) with a restricted choice and 11 (12%) offering an unrestricted choice. Among the 10 nonprofit-sponsored trials, the choice was restricted in 7 RCTs (7.6%) and unrestricted in 3 (3.3%) (Figure).

**Table.** Characteristics of Published Oncology RCTs Mentioning Physician’s Choice or Investigator’s Choice in the Control Group (N = 92)

Oncology RCT characteristics	Published oncology RCTs, No. (%) (N = 92)		
	Unrestricted choice		Overall (n = 92)
	No (n = 78)	Yes (n = 14)	
Tumor type			
Acute myeloid leukemia	3 (3.8)	1 (7.1)	4 (4.3)
All types	0	1 (7.1)	1 (1.1)
Amyloidosis	1 (1.3)	0	1 (1.1)
Breast cancer	19 (24.4)	3 (21.4)	22 (23.9)
Chronic lymphocytic leukemia	1 (1.3)	1 (7.1)	2 (2.2)
Colorectal cancer	5 (6.4)	0	5 (5.4)
Diffuse large B-cell lymphoma	1 (1.3)	0	1 (1.1)
Endometrial cancer	1 (1.3)	0	1 (1.1)
Esophageal cancer	3 (3.8)	0	3 (3.3)
Follicular lymphoma	1 (1.3)	0	1 (1.1)
Gallbladder cancer	0	1 (7.1)	1 (1.1)
Gastric cancer	4 (5.1)	0	4 (4.3)
Glioblastoma	0	2 (14.3)	2 (2.2)
Glioma	1 (1.3)	0	1 (1.1)
Head and neck squamous cell carcinoma	2 (2.6)	0	2 (2.2)
Lymphoma	1 (1.3)	0	1 (1.1)
Mantle-cell lymphoma	2 (2.6)	0	2 (2.2)
Melanoma	4 (5.1)	1 (7.1)	5 (5.4)
Mesothelioma	1 (1.3)	0	1 (1.1)
Nasopharyngeal cancer	0	1 (7.1)	1 (1.1)
Non–small cell lung cancer	7 (9.0)	1 (7.1)	8 (8.7)
Ovarian cancer	7 (9.0)	1 (7.1)	8 (8.7)
Pancreas cancer	2 (2.6)	0	2 (2.2)
Prostate cancer	2 (2.6)	0	2 (2.2)
Renal cancer	1 (1.3)	1 (7.1)	2 (2.2)
Small-cell lung cancer	2 (2.6)	0	2 (2.2)
T-cell lymphoma	3 (3.8)	0	3 (3.3)
T-cell lymphoma/leukemia	1 (1.3)	0	1 (1.1)
Urothelial carcinoma	3 (3.8)	0	3 (3.3)
Setting			
Advanced	72 (92.3)	12 (85.7)	84 (91.3)
Early	6 (7.7)	2 (14.3)	8 (8.7)
Design			
Blind	4 (5.1)	1 (7.1)	5 (5.4)
Open	74 (94.9)	13 (92.9)	87 (94.6)
Phase			
2	27 (34.6)	8 (57.1)	35 (38.0)
3	51 (65.4)	6 (42.9)	57 (62.0)
Sponsor			
Any industry involvement	71 (91.0)	11 (78.6)	82 (89.1)
No industry involvement	7 (9.0)	3 (21.4)	10 (10.9)

Abbreviation: RCT, randomized clinical trial.

**Figure.**
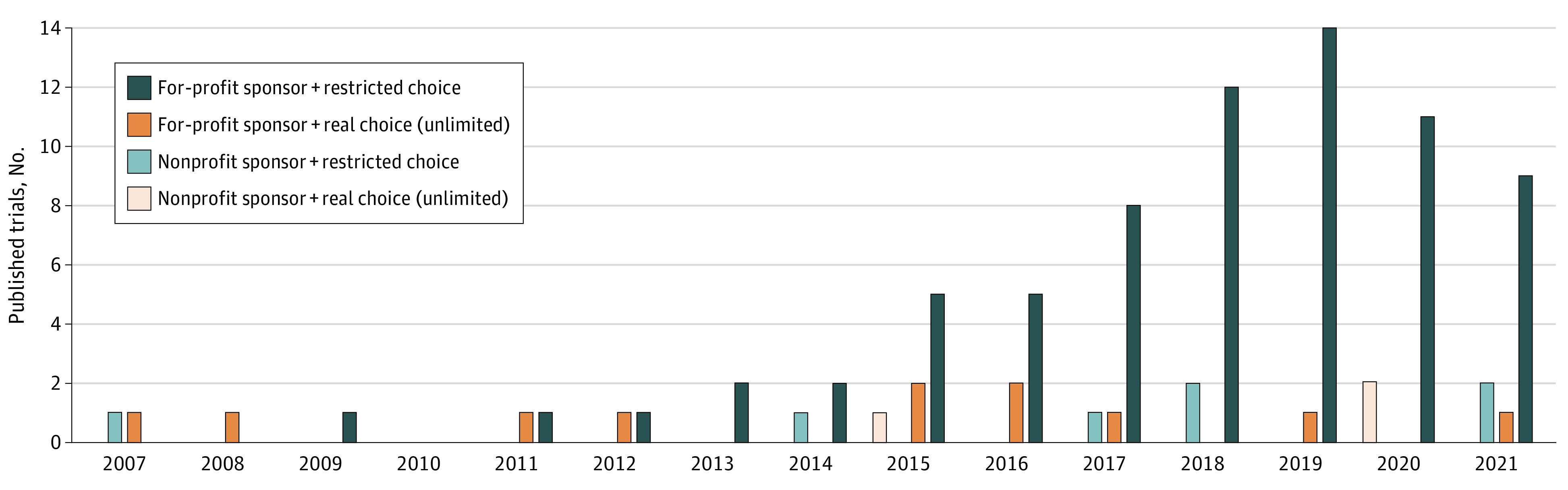
Cumulative Yearly Number of Published Oncology Randomized Clinical Trials Mentioning Physician’s Choice or Investigator’s Choice in the Control Group (N = 92)

## Discussion

This cross-sectional study, to our knowledge the first to address this research question, found that the physician’s or investigator’s choice may be ambiguously reported and is associated with a restricted choice in most oncology RCTs using these terms. Most of these reports are also funded at least partly by for-profit entities.

Consider the most represented tumor type of our included trials, breast cancer (23.9%): in a trial investigating sacituzumab govitecan in patients with triple negative breast cancer, the physician’s choice was restricted to 4 options, not allowing platinum nor anthracyclines, 2 highly active agents, possibly leading to substandard outcomes.^3^

One limitation of our study is that we did not assess for the prevalence of the included reports as compared with all published oncology RCTs. As an estimation, when they were 121 published RCTs in breast cancer between 2014 and 2017, we included 6 breast cancer trials (6 of 121; 4.6%).^2^ Also, as another potential limitation, we restricted our search to MEDLINE.

Physician’s or investigator’s choice have been used for decades in the literature; however, they are increasing over time. This is an important trend to be aware of in oncology RCTs. Through imprecise wording, potentially masking substandard control group, treating physicians may inaccurately think that the reported results can be generalized to their patients, whereas this may not be true. Our findings suggest that editors and regulators should demand clarification in the use of these terms within RCTs protocols and reports.

## Notes

Supplement. **eMethods.** Method for the Research and Selection of Articles **eFigure.** Flowchart of the Article Selection Process
