# Supplementary material for: Reporting of Physicians’ or Investigators’ Choice of Treatment in Oncology Randomized Clinical Trials
Source: JAMA Netw Open. 2022 Jan 21;5(1):e2144770. doi: 10.1001/jamanetworkopen.2021.44770 (PMC8783268; doi:10.1001/jamanetworkopen.2021.44770)
Supplement: Supplement. — eMethods. Method for the Research and Selection of Articles eFigure. Flowchart of the Article Selection Process [file jamanetwopen-e2144770-s001.pdf]

Supplemental Online Content

Olivier T, Haslam A, Prasad V. Reporting of physicians’ or investigators’ choice of treatment in oncology randomized clinical trials. *JAMA Netw Open.* 2022;5(1):e2144770. doi:10.1001/jamanetworkopen.2021.44770

eMethods. Method For the Research and Selection of Articles

eFigure. Flowchart of the Article Selection Process

This supplemental material has been provided by the authors to give readers additional information about their work.

© 2022 Olivier T et al. *JAMA Network Open.*

eMethod. Method For the Research and Selection of Articles

In PubMed website (https://pubmed.ncbi.nlm.nih.gov/), we conducted the following research:
(« physician's choice » OR « physicians' choice » OR « physician choice » OR « investigator's choice » OR « investigators' choice » OR « investigator choice ») AND (« randomized » OR « randomised »)

The research was conducted on September, 1st, 2021
All the data were coded independently by two reviewers (TO, AH).

Inclusion criteria:

- randomized trials studying anticancer intervention
- Using of the word "physician's choice" OR "investigator's choice" in either the title or the abstract

Exclusion criteria:

- Non-oncology topic
- Not assessing anticancer drug (supportive care, surgery)
- Other than single randomized trial (commentary, perspective, single-arm trial, reviews, cost-effectiveness analysis, analysis of multiples RCTs, meta-analyses, others)
- Re-analysis or subsequent publication of a trial
- Words "physician's choice" OR "investigator's choice" not used to refer to treatment arms

© 2022 Olivier T et al. *JAMA Network Open.*

eFigure. Flowchart of the Article Selection Process

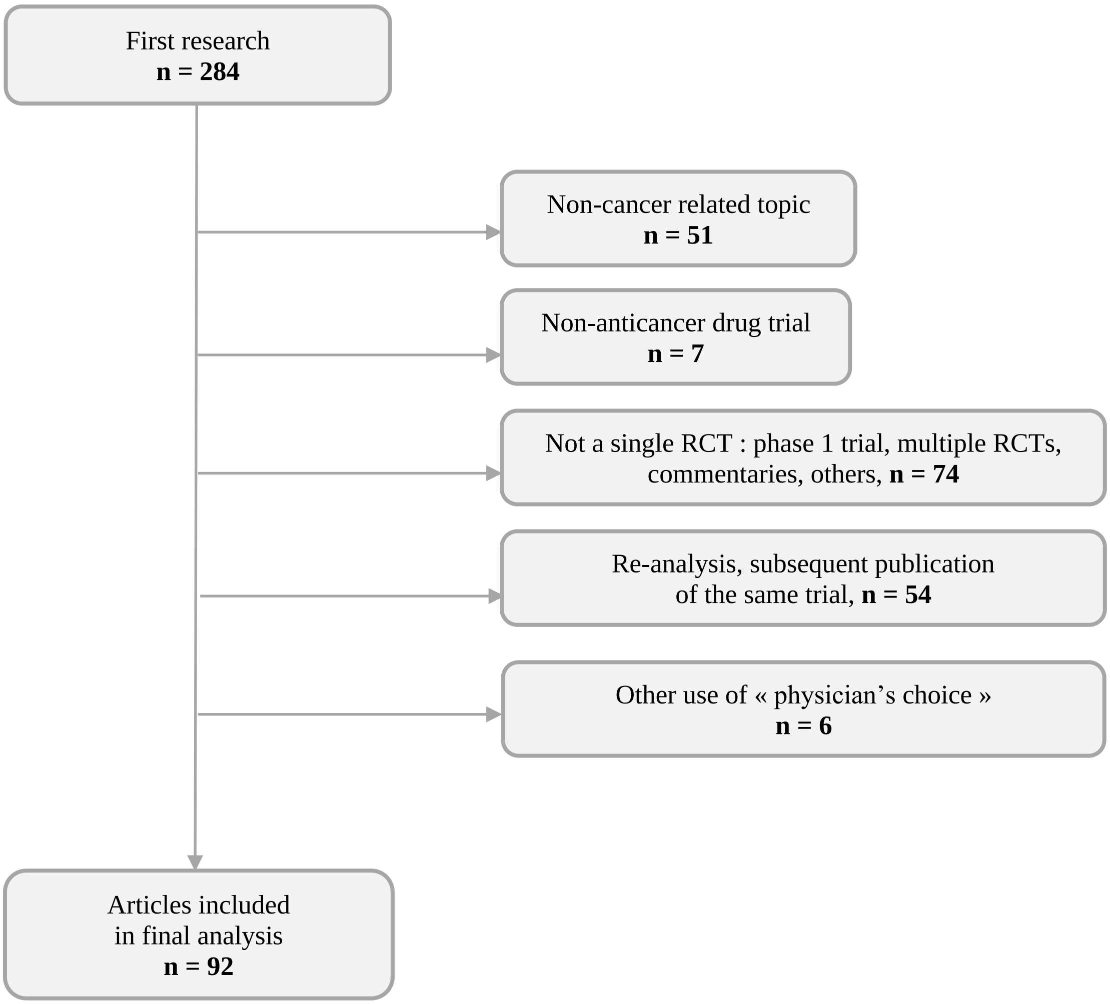

© 2022 Olivier T et al. *JAMA Network Open.*
